# High-Performance Work Systems and Well-Being: Mediating Role of Work-to-Family Interface

**DOI:** 10.5334/pb.473

**Published:** 2019-08-13

**Authors:** Audrey Babic, Florence Stinglhamber, Isabelle Hansez

**Affiliations:** 1 Human Resources Development Unit (Psychology and Educational Sciences Faculty), University of Liège, BE; 2 Psychological Sciences Research Institute (IPSY), Université catholique de Louvain, BE

**Keywords:** high performance work systems, work-to-family conflict, work-to-family enrichment, job strain, job engagement

## Abstract

Regarding the effects of High-Performance Work Systems (HPWS), we can draw two conclusions. First, existing studies on the effects of HPWS on employees’ well-being at work are scarce. Second, few studies have considered the relationships between HPWS and work-to-family interface (i.e., work-to-family enrichment, WFE; and work-to-family conflict, WFC). Only one previous study conducted on a Portuguese sample (i.e., Carvalho & Chambel, 2016) has examined the relationships between these concepts in a comprehensive model. Our study aims to replicate one part of Carvalho and Chambel’s model but also to extend previous work. We investigated a model of HPWS-employees’ well-being at work (i.e., job engagement and job strain) relationships by considering work-to-family interface as a mediator. We surveyed 170 employees of a Belgian company. Data were analysed using structural equation modelling and bootstrapping method. WFE partially mediates the relationships between HPWS and job engagement, whereas WFC partially mediates the relationships between HPWS and job strain. Our study, confirming the results of Carvalho and Chambel (2016), highlights the important role of HPWS in the development of employees’ well-being at work. Working in an organization where HPWS are applied leads employees to perceive more enrichment and less conflict between their work and family lives, making them more engaged in and less stressed by their work.

## Introduction

For several years now, a growing body of research has focused on human resource management practices labelled “high performance work systems” (HPWS). This concept refers to “a specific combination of human resource practices, work structures, and processes that maximizes employee knowledge, skill, commitment and flexibility” (Bohlander & Snell, 2007, p. 690). These practices are interconnected and designed to increase employees’ competencies and motivation and to enhance employees’ and organizations’ performance (Appelbaum, Bailey, Berg & Kalleberg, 2000), thereby contributing to organizations’ competitive advantages (Combs, Liu, Hall, & Ketchen, 2006).

When looking at the effect of HPWS, some interesting points emerge. Firstly, regarding the effects on employees’ work-related well-being, the currently limited data available on the topic reports mixed findings. Some researchers have found a positive effect on employee well-being. For example, HPWS were found to contribute to employees’ well-being, especially by enhancing their sense of value, worth, and security (Wood & de Menezes, 2011), increasing job engagement (Mihail & Kloutsiniotis, 2016; Zhang, Cherrie, Dowling & Bartram, 2013), and decreasing burnout (Fan, Cui, Zhang, Zhu, Hartel, & Nyland, 2014) and job strain (Wood, Van Veldhoven, Croon & de Menezes, 2012). However, other scholars have adopted a negative view of the influence of HPWS on employees’ work-related well-being. They found that, by intensifying job demands, HPWS practices lead to stress or emotional exhaustion (Ehrnrooth & Björkman, 2012; Godard, 2001; Kroon, Van de Voorde & Van Veldhoven, 2009; Ramsay, Scholarios & Harley, 2000).

Secondly, few studies have investigated the relationships between HPWS and work–to-family interface, i.e., work-to-family enrichment (WFE - the extent to which experiences at work improve the quality of individuals’ family lives; Greenhaus & Powell, 2006) and work-to-family conflict (WFC—the extent to which work demands impede individuals’ performance in their family responsibilities; Netemeyer, Boles & McMurrian, 1996). Here again, mixed findings were reported. For example, some scholars found that HPWS (or some high involvement HR practices) allowed workers to manage their work and family responsibilities better, leading to increased perceptions of WFE (Berg, Kalleberg, & Appelbaum, 2003; Carvalho & Chambel, 2014) and reduced perceptions of WFC (Batt & Valcour, 2003). However, other scholars reported positive relationships between HPWS and WFC (Shih, Chiang & Hsu, 2010; White, Hill, McGovern, Mills, & Smeaton, 2003).

Thirdly, to the best of our knowledge, only one study, that of Carvalho and Chambel (2016), has examined the relationships of HPWS practices to subjective well-being (i.e., satisfaction with life and health perceptions) in a comprehensive model by considering work–to-family interface and well-being at work (i.e., burnout and work engagement) as serial mediators. Through a cross-sectional sample of 218 employees of a Portuguese city council, they found (1) positive relationships between perceived HPWS and WFE, (2) negative relationships between perceived HPWS and WFC, and (3) work-to-family interface and well-being at work acting as serial mediators.

Based on these observations and considering the importance of allowing workers to manage their work and family lives advantageously and to promote their well-being at work (Andersen, Proper, Punnett, Wynne, Persson & Wiezer, 2015), the present study aims to investigate the relationships between HPWS, work-to-family interface and well-being at work in an attempt to replicate part of Carvalho and Chambel’s (2016) results. The present study, thus, hopes to confirm the results from the only research investigating these relationships but also to respond to Guest’s recommendations (2011) (i.e., to better understand the theoretical mechanisms underlying the HPWS-employee’s work-related well-being relationships) and to those of Carvalho and Chambel (2014) (i.e., to better understand the effects of HPWS practices on work–family interface). Moreover, focusing on employees’ viewpoints and examining the employees’ outcomes of HPWS, this study also intends to respond to the call for more research on HPWS from an employee’s perspective (e.g., Van De Voorde & Beijer, 2015). Indeed, whereas positive effects of HPWS on organizational outcomes such as performance or productivity are well established, fewer studies have investigated the influence of HPWS on employees’ outcomes (Zhang & Morris, 2014).

Based on Carvalho and Chambel’s (2016) findings, we consistently adopted a positive view of the influence of HPWS. However, there are some distinctions between the study of Carvalho and Chambel and ours, notably in the sample and the concepts used to measure the negative indicator of well-being at work. Moreover, we extend Carvalho and Chambel’s work in two ways. First, we considered the relationships between(1) WFE and positive well-being at work (i.e., job engagement), (2) WFC and negative well-being at work (i.e., job strain) but also (3) WFE and job strain, and (4) WFC and job engagement. We do so following the recommendation of Peeters, ten Brummelhuis, and van Steenbergen, (2013) to further analyse the impact of WFC/WFE on well-being, and also because literature on the relationships between WFE and negative indicators of well-being is scarce (Peeters et al., 2013). Secondly, given that the design used by Carvalho and Chambel (2016) was cross-sectional in nature, we reflected on whether alternative causal orderings were possible by considering well-being at work as a mediator in the relationships between HPWS and work-to-family interface.

### High-Performance Work Systems and well-being at work: The mediating role of work-to-family interface

High-Performance Work Systems (HPWS) are comprehensive bundles of practices aimed at managing employees in organizations. These practices interact to “select, develop, and motivate a workforce that has outstanding qualities and that uses these qualities in work-related activities with discretionary effort, which result in improved organizational performance and sustained competitive advantage for the organization” (Appelbaum et al., 2000, cited by Kroon et al., 2009, p. 510). Such practices are “designed to enhance employees’ competencies, motivation, opportunities to contribute, and consequently engender employee and organizational performance” (Datta, Guthrie, & Wright, 2005; Huselid, 1995; Lepak, Liao, Chung, & Harden, 2006; Way, 2002; Wright & Snell, 1991, cited by Chang & Chen, 2011, p. 883). However, even if there is no standard list of the components of HPWS, it is fairly well established that these practices should be multiple and mutually reinforcing (Cappelli & Neumark, 2001; Wood & Wall, 2001). Moreover, Godard (2004) has argued that the benefit of HPWS increases with the number of practices. As mentioned by Becker and Huselid (1998, p. 63), “the overwhelming preference in the literature has been for a unitary index that contains a set (though not always the same set) of theoretically appropriate human resource management practices derived from prior work.” Therefore, considering HPWS as a single system is theoretically appropriated.

Some scholars argue that HPWS have a positive influence on the way in which workers manage their work and family responsibilities. Indeed, organizations characterized by HPWS tend to use more family-friendly practices (e.g. flexible working time or career-break practices; e.g., Berg et al., 2003; Wang & Verma, 2012) in comparison to traditional organizations, especially to increase their workers’ levels of commitment. These family-friendly practices allow workers to better manage work and family lives, leading them to perceive lower levels of WFC and higher levels of WFE (e.g., Berg, Kalleberg, & Appelbaum, 2003; Frye & Breaugh, 2004; Osterman, 1995).

Moreover, HPWS practices can be considered as job resources (Saks & Rotman, 2006; Schaufeli & Bakker, 2004). Indeed, HPWS practices allow employees to learn from their colleagues, enhance personal development, acquire new skills, or be involved in a more meaningful job (Loughlin & Mercer, 2014). By providing or increasing job resources, HPWS promote employees’ sense of personal control and efficacy at work, improving their capacities to manage work and family responsibilities (Greenhaus & Beutell, 1985; Voydanoff, 1988). Resources are crucial to manage work and family spheres effectively, which means to perceive enrichment and avoid conflict. Indeed, according to the enrichment process (Greenhaus & Powell, 2006), resources gained from the work domain can be transferred to the family, through two different paths, thereby improving individuals’ quality of life in their private domain. The first path, the *instrumental path* (direct transfer), explains how skills, behaviours and rewards from work (acquired for example through HPWS) can help individuals function better in the family. In other words, the work environment creates a sense of personal control, self-esteem, self-efficacy, self-confidence and psychological resources (Greenhaus & Powell, 2006). These perceptions have positive impacts on workers’ ability to manage their work and family lives, leading them to perceive WFE (Greenhaus & Beutell, 1985; Voydanoff, 1988). The second path, the *affective path*, refers to the degree to which mood and emotions from work can positively impact how individuals feel, act and behave within the family. Here, the positive influence between domains is indirect; a resource acquired at work through HPWS engenders positive emotions at work, which in turn improves individual functioning in the family (Greenhaus & Powell, 2006).

Empirical studies provide evidence that working in an environment applying HPWS allows workers to better manage their work and family responsibilities. Through a sample of 557 dual-earner white collar employees, Batt and Valcour (2003) investigated the relationships between some high involvement HR practices which are close to HPWS (Appelbaum et al., 2000) and three outcomes including, notably, WFC. Their results clearly showed both job security and supportive supervision negatively related to WFC. In a sample of employees from a Portuguese bank, Carvalho and Chambel (2014) found a positive relationship between HPWS and WFE. Two years later, they found that HPWS related positively with WFE and negatively with WFC (Carvalho & Chambel, 2016).

In terms of well-being, it is reasonable to assume that efficiently managing the demands of workers’ various roles and, therefore, handling work and family responsibilities well, could influence two indicators of employees’ work-related well-being (Brauchli, Schaufeli, Jenny, Füllemann, & Bauer, 2013), namely job engagement and job strain.

Indeed, based on the COR theory (Hobfoll, 2002), when employees perceive that their work provides something (i.e., resources) beneficial to themselves or their families (i.e., WFE), they want to obtain more of such resources and, consequently, they are more engaged in their job. This theory also assumes that people possessing resources (i.e., perceiving WFE) are less likely to see their well-being negatively influenced by stressful circumstances, consequently leading them to experience less stress at work. Thus, “when not currently confronted with stressors” (i.e., a situation of WFE), “people strive to develop resource surpluses in order to offset the possibility of future loss” and in doing so “they are likely to experience positive well-being” (Hobfoll, 1989, p. 517).

In contrast, situations where workers perceive a loss of resources arising from the juggling of work and family roles (i.e., inter-role conflict situations) generate stress (Hobfoll, 2002). Indeed, stress is viewed as “a reaction to the environment in which there is (a) the threat of a net loss of resources, (b) the net loss of resources, or (c) a lack of resource gain following the investment of resources. Both perceived and actual loss or lack of gain are envisaged as sufficient for producing stress.” (Hobfoll, 1989, p. 516). When facing a stressful situation of WFC (i.e., a situation of resource depletion), workers tend to protect or limit the loss of their remaining resources (Hobfoll, 2002), notably by reducing their engagement in their work, given that work impedes employees’ functioning in the family domain and leads to loss of resources.

Empirical studies support these contentions. For example, Wayne, Musisca, and Fleeson (2004) found that when people experience WFE, they report making greater efforts in their job by increasing their engagement with work. Carvalho and Chambel (2016) also highlighted this positive WFE-job engagement relationship. In a sample of social workers, Kallaith (2014) found that WFE was negatively related to psychological strain. Opie and Henn’s (2013) study of 267 South African working mothers from several organizations found that employees experiencing WFC were less engaged in their work. In a sample of Belgian hospital employees, Babic, Stinglhamber and Hansez (2015) found a positive relationship between WFC and job strain.

Based on the aforementioned, and by adopting the positive viewpoint emerging from the study of Carvalho and Chambel (2016), we hypothesize that HPWS are positively related to WFE, which in turn is positively related to workers’ job engagement and negatively related to job strain. We also postulate that HPWS are negatively related to WFC, which in turn is positively related to workers’ job strain and negatively related to job engagement. In other words, we establish through the first part of our hypothesis that:

*Hypothesis 1a:* WFE mediates the relationships between HPWS and well-being at work.

*Hypothesis 1b:* WFC mediates the relationships between HPWS and well-being at work.

### High-Performance Work Systems and work-to-family interface: The mediating role of well-being at work

The reserve causation, i.e., the fact that HPWS practices are related to well-being at work, which in turn is related to work-to-family interface, is also supported theoretically. As previously mentioned, components of HPWS practices can be considered as job resources (Saks & Rotman, 2006; Schaufeli & Bakker, 2004). The JD-R model (Schaufeli & Bakker, 2004) argues that job resources lead to greater job engagement through a motivational process (Bakker & Demerouti, 2007). According to Schaufeli and Bakker (2004, p. 298), job resources “may play either an intrinsic motivational role because they foster employees’ growth, learning, and development, or they may play an extrinsic motivational role because they are instrumental in achieving work goals.” In the same vein, in line with COR theory (Hobfoll, 2002), individuals seek to acquire and maintain resources. In situations that provide prospects for enhancing resources (i.e., an environment applying HPWS), individuals will be motivated to make an effort and even persist in difficult situations, because success will provide the expected gain in resources. Moreover, individuals possessing resources are more able to face stressful situations, and, therefore, less likely to experience negative outcomes, such as job strain (Hobfoll, 2002).

Accordingly, it is reasonable to assume that, by giving access to job resources and fostering the development of personal resources, HPWS practices lead workers to be more engaged in work and perceive less job strain. Here again, empirical studies support these arguments. For example, Mihail and Kloutsiniotis, (2016) and Zhang et al., (2013) confirmed that HPWS were positively related to job engagement. Several studies found that the implementation of HPWS decreases workers’ job strain (Harley, Allen, & Sargent, 2007; Macky & Boxall, 2008). Some HPWS practices (e.g., teamwork) increase social contact, and other practices (e.g., information sharing) reduce uncertainty in the work environment, thereby decreasing job anxiety and job strain (Wood et al., 2012). Through involvement in the organization (i.e., teams and decentralized decision-making), workers feel they are respected and considered by their organization, which increases their self-esteem and decreases their psychological strain (Macky & Boxall, 2007).

Theoretical arguments also exist to consider the effects of well-being at work on work-to-family interface. On one hand, individuals working with enthusiasm and energy may develop and acquire new resources (i.e., skills, positive emotions, and improved self-esteem; COR theory, Hobfoll, 2002). Indeed, engagement in work may lead to a gain spiral of resources in which employees acquire more and more resources, allowing them to fulfil their work and family responsibilities more effectively. Moreover, by being engaged in or feeling more absorbed in their jobs, individuals are likely to have more positive affect that then spills over into their family life (Edwards & Rothbard, 2000), influencing affect at home and facilitating positive or beneficial interactions. On the other hand, being exposed to strain in a given domain (e.g., work) may lead to tension, irritability, fatigue, or preoccupation with problems (Greenhaus & Beutell, 1985). This negative state may spill over (Pleck, 1977), affecting an individual’s ability to function in another domain (e.g., family), leading him/her to perceive greater inter-role conflict and less enrichment. Empirical findings support these theoretical arguments. For example, in their two-wave study (but without repeated measures), Siu et al. (2010) found that job engagement was positively related to WFE. In a cross-sectional study among Finnish judges, Hakanen, Perhoniemi and Rodríguez-Sánchez (2012) discovered that job engagement was negatively related to WFC. Matthews, Wayne and Ford (2014) found that greater subjective well-being was associated with reduced WFC over time. In their meta-analysis focusing on 32 studies based on cross-lagged panel designs, Nohe, Meier, Sonntag and Michel (2015) highlighted the positive effects of work-specific strain (e.g., disengagement, emotional exhaustion, irritation, need for recovery, and personal accomplishment) on WFC.

Based on the aforementioned, we establish that HPWS are positively related to job engagement, which in turn is positively related to WFE and negatively related to WFC. We also postulate that HPWS are negatively related to job strain, which in turn is positively related to WFC and negatively related to WFE. In other words, in the second part of our hypothesis, we suggest that:

*Hypothesis 2a:* Job engagement mediates the relationships between HPWS and work-to-family interface.

*Hypothesis 2b:* Job strain mediates the relationships between HPWS and work-to-family interface.

## Methods

### Sample and procedure

An electronic questionnaire was administered to employees from a Belgian inter-municipal company serving a considerable number of local communities. This company’s mission is to work in the public interest to improve the welfare of residents living in the region. Four hundred fifty-five individuals were invited to participate in this research. They had three weeks to complete the anonymous and confidential questionnaire. We received 170 questionnaires in return, a response rate of about 37%. Most of the participants were male (72%), on average, 40.51 years old (*SD* = 9.86). Over one third of the respondents (35%) had been employed by their company for 11 to 20 years. These socio-demographic variables were unrelated to our model’s constructs and, consequently, were not used as control variables (Little, 2013).

### Measures

*High-Performance Work Systems* (HPWS) were measured with the questionnaire developed by Zacharatos, Barling, and Iverson (2005). This emphasized employees’ perceptions of the extent to which the organization had adopted ten human resource practices: *Employment Security* (e.g., “I can be sure of being employed in my organization as long as I do good work”); *Information Sharing* (e.g., “It is easy for me to communicate my thoughts to management”); *Selective Hiring* (e.g., “Only the best are hired to work in my organization”); *Training* (e.g., “The company provides enough training for me to learn new ways to do my job”); *Teams and Decentralized Decision-Making* (e.g., “If there is a decision to be made, everyone is involved in it”); *Reduced Status Distinctions* (e.g., “I have the opportunity to interact with top management in my organization”); *Contingent Compensation* (e.g., “Part of my compensation is based on how well my workgroup or department performs”); *Transformational Leadership* (e.g., “My supervisor treats each of us as individuals with different needs, abilities and aspirations”); *Job Quality* (e.g., “I have lots of opportunity to decide how to do my work”); and *Measurement* (e.g., “This organization tries to find out how its employees are feeling”). Each scale comprised five items, except *information sharing*, which included six. All items were answered using a 5-point Likert-type scale ranging from 1 (strongly disagree) to 5 (strongly agree).

According to a fundamental principle of strategic human resource management research, the impact of human resource practices is best understood by examining the system of practices as a whole, rather than examining individual practices (e.g., Guthrie, 2001; Wright & Boswell, 2002). Thus, following Drasgow and Kanfer’s (1985) and Zacharatos et al.’s (2005) recommendation, we applied the subscale aggregation approach. We calculated the subscale scores, averaging across items of the same practice dimension. The ten subscale scores obtained represent the observed variables which constituted indicators of our HPWS latent factor.

*Work-to-family conflict* (WFC) and *work-to-family enrichment* (WFE) were measured using the validated French version of the two ad hoc subscales of the Survey Work-Home Interaction–Nijmegen (Hansez, Etienne, & Geurts, 2006). The WFC subscale contains nine items (e.g., “I’m irritable at home because my work is demanding”). The WFE subscale includes six items (e.g., “I come home cheerfully after a successful day at work, positively affecting the atmosphere at home”). Individuals respond using a 4-point Likert-type scale (0: never to 3: always).

*Job strain* was measured with the Negative Occupational State Inventory subscale (NOSI) and *job engagement* with the Positive Occupational State Inventory subscale (POSI) developed by Barbier, Monseur, Bertrand and Hansez (2012). Both have been used in diverse occupational fields (e.g., Babic et al., 2015; Babic, Stinglhamber, Bertrand, & Hansez, 2017). The NOSI subscale comprises eleven items (e.g., “I feel demoralized by my work”). The POSI subscale comprises eight items (e.g., “I’m full of energy at work”). For each subscale, respondents answered using a 4-point Likert-type scale (1: never to 4: always).

Questionnaires were sent in French. Therefore, we translated originally English written scales following a translation back-translation procedure. They were first translated from English to French, and then back-translated from French to English by a native speaker. For all scales, there was no major discrepancy between the original and translated English versions of the scales, so the translation process was considered appropriate.

### Data analyses

Structural equation modelling analyses (SEM) were performed using LISREL 8.80 (Jöreskog & Sörbom, 2006). We analysed data following a two-stage process (Anderson & Gerbing, 1988). Firstly, we assessed the measurement model through a series of confirmatory factor analyses to evaluate the independence of the constructs we examined. Second, we proceeded with the assessment of the hypothesized structural relationships among latent variables. We also used the bootstrapping technique to estimate indirect effects (Preacher & Hayes, 2008). Bootstrapping analyses were conducted with the PROCESS macro (Hayes, 2013).

Based on the confirmatory factor analyses and by using the balancing technique (Little, Cunningham, Shahar & Widaman, 2002), we reduced to three the number of indicators for WFC, WFE, job strain and job engagement, using a parcelling strategy (Little, Rhemtulla, Gibson & Schoemann, 2013). We proceeded in this way for two main reasons. Firstly, given the small size of our sample, parcels allow us to limit the number of parameters to be estimated (Landis, Beal, & Tesluk, 2000). Secondly, we used parcels to maintain the robustness of the analysis and preserve common construct variance while minimizing unrelated specific variance (Little et al., 2002).

## Results

### Discriminant validity

We compared several nested models to test the distinctiveness of the constructs included in our study (Bentler & Bonett, 1980). First, we examined the fit of our hypothesized five-factor model that comprises HPWS, WFE, WFC, job strain and job engagement, and also a series of more constrained measurement models. We used chi-square difference tests to compare the fit of these nested models with that of the five-factor model (Bentler & Bonett, 1980). Results indicated that the five-factor model was significantly superior to any alternatives models. Consequently, we treated these five constructs as independent from each other in subsequent analyses. Table 1 displays fit indices of some of these alternative models.

**Table 1:** Fit indices for measurement models.

Model	*χ*^2^	*df*	*χ*^2^/*df*	NNFI	CFI	RMSEA	Δ*χ*^2^ (Δdf)

5-factor model	317.50	199	1.59	.97	.97	.06	–
4-factor model: HPWS with job strain	453.75	203	2.23	.94	.95	.09	136.26 (4)***
4-factor model: HPWS with job engagement	459.07	203	2.26	.94	.95	.09	141.58 (4)***
4-factor model: WFC with job strain	438.94	203	2.16	.95	.95	.09	121.44 (4)***
4-factor model: WFE with job engagement	406.41	203	2.00	.95	.96	.08	88.92 (4)***
4-factor model: WFC with WFE	542.69	203	2.67	.89	.93	.10	225.19 (4)***
4-factor model: job strain with job engagement	534.43	203	2.63	.92	.93	.11	216.93 (4)***
3-factor model: WFC with WFE; job strain with job engagement	746.31	206	3.62	.87	.89	.15	428.81 (7)***
1-factor model	1001.93	209	4.79	.82	.83	.17	684.43 (10)***

*Note*: *N* = 170. HPWS = high-performance work systems; WFC = work-to-family conflict; WFE = work-to-family enrichment; χ^2^ = Minimum Fit Function Chi-Square; df = degrees of freedom; NNFI = Non-Normed Fit Index; CFI = Comparative Fit Index; RMSEA = root-mean-square error of approximation; Δχ^2^ = chi-square difference tests between the five-factor model and alternative models. *** *p* < .001.

### Relationships among variables

Means, standard deviations, Cronbach’s alphas and correlations among variables are presented in Table 2. Internal consistency reliabilities ranged from .81 to .94.

**Table 2:** Descriptive statistics and inter-correlations among variables.

	Variables	*M*	*SD*	1	2	3	4	5	6	7	8

1	Gender	–	–	–							
2	Age	40.51	9.86	–.14	–						
3	Organizational tenure	–	–	–.05	.55***	–					
4	High-Performance Work Systems	3.09	.53	–.10	.00	.02	(.94)				
5	Work-to-family conflict	.71	.55	–.08	.06	.00	–.25***	(.88)			
6	Work-to-family enrichment	.91	.56	–.07	.01	–.03	.39***	–.15	(.81)		
7	Job engagement	2.73	.57	–.06	.03	.03	.59***	–.12	.52***	(.83)	
8	Job strain	1.80	.46	–.07	.07	.04	–.55***	.56***	–.17*	–.31***	(.82)

*Note*: *N* = 170. Correlations among variables are provided below the diagonal and Cronbach’s alphas are provided on the diagonal. Absence of means and standard deviations for gender and organizational tenure because the answers were beforehand categorized in the questionnaire. * *p* < .05, *** *p* < .001.

Starting from this five-factor model, we compared several competitive structural models (Table 3). In all models, we allowed disturbance terms of outcomes to correlate. First, we compared two competitive structural models (i.e., Model A and B) to assess the causal relationships between variables. Model A (Hypotheses 1a and 1b) included indirect paths from HPWS to well-being (i.e., job engagement and job strain) through work-to-family interface (i.e., WFE and WFC) (ECVI = 2.42). Model B (Hypotheses 2a and 2b) included indirect paths from HPWS to work-to-family interface through well-being (ECVI = 2.71). The comparison of ECVI suggested that Model A better represented the relationships among constructs. These results did not support Hypotheses 2a and 2b. Therefore, we referred to Model A as the retained structural model (Model 1).

**Table 3:** Fit indices for structural models.

Model	*χ*^2^	*df*	*χ*^2^/*df*	NNFI	CFI	RMSEA	ECVI	Comparison	Δ*χ*^2^ (Δdf)

Model A (Model 1): Hypothesized theoretical model HPWS → work-to-family interface → well-being	391.01	202	1.94	.95	.96	.07	2.42	Model 1 VS Model 2	35.42(1)***
Model B: Hypothesized theoretical model HPWS → well-being → work-to-family interface	397.79	202	1.97	.92	.93	.08	2.71	Model 1 VS Model 3	73.14(2)***
Model 2: Model 1 + Paths between HPWS and job strain	355.59	201	1.77	.96	.97	.06		Model 2 VS Model 3	37.72(1)***
Model 3: Model 2 + Paths between HPWS and job engagement	317.87	200	1.59	.97	.98	.06		–	–

*Note*: *N* = 170. HPWS = high-performance work systems; χ^2^ = Minimum Fit Function Chi-Square; df = degrees of freedom; NNFI = Non-Normed Fit Index; CFI = Comparative Fit Index; RMSEA = root-mean-square error of approximation; Δχ^2^ = chi-square difference tests between the five-factor model and alternative models. *** *p* < .001.

Model 1 fitted the data reasonably well, as indicated by the following indices: χ^2^ (202) = 391.01, *p* < .001, RMSEA = .07, NNFI = .95, CFI = .96. To evaluate whether Model 1 offered the best depiction of our data, we compared it with two alternative nested models containing additional theoretically plausible paths. Starting from Model 1, we added a path from HPWS to job strain (Model 2). This Model 2 fitted the data reasonably well (χ^2^ (201) = 355.59, *p* < .001, RMSEA = .06, NNFI = .96, CFI = .97). The chi-square difference test indicated that Model 2 was significantly superior to Model 1 (Δχ^2^(1) = 35.42, *p* < .001). Starting from Model 2, we added a path from HPWS to job engagement (Model 3). This Model 3 fitted the data reasonably well (χ^2^ (200) = 317.87, *p* < .001, RMSEA = .06, NNFI = .97, CFI = .98). Results of the chi-square difference test indicate that Model 3 was significantly superior to Model 2 (Δχ^2^(1) = 37.72, *p* < .001). We thus retained Model 3 as the best fitting model.

**Figure 1:**
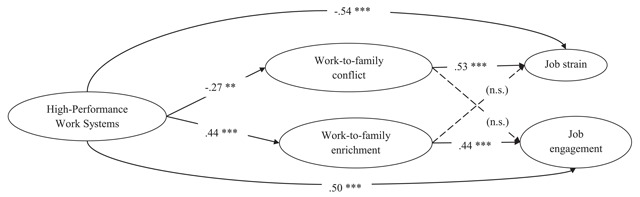
Completely standardized path coefficients for the retained model (model 3). For the sake of clarity, only structural relationships are shown. ** *p* < .01; *** *p* < .001.

Standardized parameter estimates for this model are shown in Figure 1. For ease of presentation, we show the structural model rather than the full measurement model. On the one hand, HPWS was positively associated with WFE which, in turn, was only related positively to job engagement. HPWS was also directly and positively related to job engagement. Results of the bootstrap analyses indicated that the indirect effect of HPWS on job engagement through WFE was significant (indirect effect = .14; BCa 95% CI = [08; .23]). Thus, WFE partially mediated the effects of HPWS on job engagement. These findings partially supported Hypothesis 1a, given that the WFE-job strain relationship was non-significant.

On the other hand, HPWS was negatively related to WFC which, in turn, was only positively related to job strain. HPWS was also directly and negatively related to job strain. The indirect effect of HPWS on job strain through WFC was significant (indirect effect = –.10; BCa 95% CI = [–.20; –.03]). Consequently, WFC partially mediated the effects of HPWS on job strain. These findings partially supported Hypothesis 1b, given that the WFC-job engagement relationship was non-significant.

## Discussion

This study’s aim was to confirm the results of the only comprehensive study on relationships between HWPS, work-to-family interface and well-being at work. Our research tends to replicate one part of Carvalho and Chambel (2016)’s results but also extends their work by investigating the relationships between WFC and positive indicator of well-being at work (i.e., job engagement), and between WFE and negative indicator of well-being at work (i.e., job strain). We also investigate an alternative causal model by considering well-being at work as a mediator in the HPWS-work-to-family interface relationships. We postulated that WFE (Hypothesis 1a) and WFC (Hypothesis 1b) mediated the relationships between HPWS and well-being at work. We also hypothesized that job engagement (Hypothesis 2a) and job strain (Hypothesis 2b) mediated the relationships between HPWS and work-to-family interface. Results partially support Hypotheses 1a and 1b and do not support Hypotheses 2a and 2b. Our study reveals that WFE and WFC partially mediate the relationships between HPWS and, respectively, job engagement and job strain. In other words, working in an HPWS environment is (a) positively related to WFE which is, in turn, only positively associated with job engagement; and (b) negatively related to WFC which is, in turn, only positively associated with job strain.

As expected, HPWS showed positive relationships with WFE and negative relationships with WFC. The fact that organizations implementing HPWS include family-friendly practices as a human resources strategy (Berg et al., 2003; Osterman, 1995) allows workers to better integrate their work and family lives, reducing their perception of WFC (Batt & Valcour, 2003; Frye & Breaugh, 2004) while increasing their perception of WFE (Carvalho & Chambel, 2014). By implementing HPWS within their organization, employers also provide employees with job resources (Carvalho & Chambel, 2014; Loughlin & Mercer, 2014; Saks & Rotman, 2006; Schaufeli & Bakker, 2004) allowing them to better manage their work and family lives. Indeed, according to the enrichment process (Greenhaus & Powell, 2006), resources gained from work (i.e., skills, behaviours, rewards, emotions, and moods) are directly or indirectly transferred to the family sphere and, thereby, lead individuals to function better in this domain. A work environment providing resources also generates a sense of personal control, and increases self-esteem, self-efficacy, and self-confidence, allowing workers to better manage work and family (Greenhaus & Powell, 2006).

As postulated, WFC showed a positive relationship with job strain, whereas WFE was positively related to job engagement. Consistent with COR theory (Hobfoll, 1989, 2002), a loss of resources due to juggling work and family roles (i.e., a perception of WFC) leads to a negative state of being for employees and a perception of higher job strain. Because role conflicts hinder personal growth or gain, they engender negative emotions and job strain (Babic et al., 2015; Gareis, Barrett, Ertel, & Berkman, 2009). People are motivated to acquire resources (COR theory; Hobfoll, 2002). Therefore, in perceiving that their work provides resources, which facilitate their functioning within the family (i.e., perception of WFE), workers tend to engage more in their jobs to continue to obtain such resources.

However, surprisingly, we found two non-significant relationships between work-to-family interface and well-being at work. On one hand, the non-significant relationship between WFE and job strain is perhaps due to the fact that we consider WFE as an overall construct rather than as a three-dimensional concept (i.e., development, affect, and capital; see Carlson, Kacmar, Wayne, & Grzywacz, 2006). Indeed, Kallaith (2014) found that WFE was negatively related to psychological strain. More precisely, her results showed that only two of the three dimensions of WFE (i.e., WFE-affect and WFE-capital) were significantly associated with reduced psychological strain. On the other hand, the non-significant relationship between WFC and job engagement could eventually be explained by the very low score of WFC (i.e., mean of .71, range = 0–3). Indeed, in their study, Wilczek-Ruzyczka, Basinska and Daderman (2012) found WFC negatively related to engagement only when WFC was high. Future studies should explore these issues further.

In addition, our results show that HPWS were directly positively related to job engagement and negatively related to job strain. Therefore, according to the motivational process of the JDR model (Schaufeli & Bakker, 2004), working in an organization applying HPWS, where job resources are available, provides both intrinsic and extrinsic motivation to employees who are, consequently, more engaged in their job. Similarly, in line with COR theory (Hobfoll, 2002), in an environment providing resources (an organization applying HPWS), individuals are motivated to make certain efforts to maintain these resources. By increasing work’s meaningfulness, manageability, and comprehensibility, high-performance work practices improve employees’ sense of coherence and their ability to manage stress, reducing their perception of job strain.

Our findings are consistent with some research studying the relationships between HPWS and work-to-family interface (e.g., Batt & Valcour, 2003; Carvalho & Chambel, 2014), between work-to-family interface and employees’ work-related well-being (e.g., Amstad, Meier, Fasel, Elfering & Semmer, 2011; Babic et al., 2015; Wayne et al., 2004), and between HPWS and employees’ work-related well-being (Mihail & Kloutsiniotis, 2016; Wood et al., 2012; Zhang et al., 2013).

Our results and those of Carvalho and Chambel (2016) diverge on one point. While we too find that WFE partially mediates the relationships between HPWS and positive indicators of well-being at work (i.e., job engagement), conclusions concerning the mediating role of WFC between HPWS and negative indicators of well-being at work differ. Carvalho and Chambel found, as we do, that HPWS reduce the perception of WFC, which in turn increases the perception of the negative indicator of well-being at work (burnout in Carvalho and Chambel’s study). However, they found a non-significant link between HPWS and the negative indicator of well-being at work, whereas we found a significant negative one between HPWS and job strain. Therefore, WFC acts as a total mediator in Carvalho and Chambel’s study, and plays a partial mediating role in our study. This difference in terms of the conclusion is, perhaps, due to the fact that burnout, the concept used by Carvalho and Chambel to assess the negative indicator of well-being at work, is viewed as “a consequence of long-term stress at work (Innstrand et al., 2008)” (Carvalho & Chambel, 2016, p. 125). Therefore, we could presume that, given the cross-sectional design of Carvalho and Chambel’s study, the authors were unable to assess the HPWS-well-being at work relationships over the long-term.

This study contributes at several levels. Firstly, this research reveals that implementing HPWS leads workers to manage the interface between work and family effectively, therefore, positively impacting their well-being at work. Consequently, this conclusion supports Carvalho and Chambel’s study (2016). Moreover, our study confirms that these positive relationships emerge in other samples (i.e., in our study, a Belgian inter-municipal company’s employees; and a Portuguese city council’s employees in Carvalho and Chambel’s study). Secondly, our study responds to recommendations concerning the need to better understand (1) the theoretical mechanisms underlying the HPWS-well-being relationship (Guest, 2011; Wood & de Menezes, 2011) and (2) the effects of HPWS practices on the work–family interface (Carvalho & Chambel, 2014). Thirdly, our study adopts an employees’ viewpoint and investigates the employees’ outcomes of HPWS. In doing so, we try to “restore the effects of HRM on employees to a central position of HPWS studies” (Gulzar, Moon, Attiq, & Azam, 2014, p 718).

### Limitations, strengths, and future perspectives

Our study has several limitations, leading us to interpret the findings reported here with caution. The first major limitation of our study is its cross-sectional design. Second, the specificity of our data (obtained from a Belgian inter-municipal company’s employees) makes it difficult to generalize our results to other professional sectors. Even if, as previously mentioned, our study confirms Carvalho and Chambel’s results (2016), our sample and theirs are both in the public sector. There are probably differences in the ways people employed in private- and public-sector organizations perceive their work. Indeed, according to Mihail and Kloutsiniotis (2016, p. 425), “the context in which organizations operate may limit or enhance the HPWS usefulness due to differences in cultural and institutional factors, that are considered country contingent, and which shape employment relationships.” Thirdly, as we used self-reported data, common-method variance may have biased our results (Podsakoff, MacKenzie, & Podsakoff, 2012). However, considering that the single-factor model showed a poor fit to the data, this common method bias was partially treated (i.e., Harman’s single-factor test; Podsakoff et al., 2012). Furthermore, as recommended by Podsakoff et al. (2012) and Richardson, Simmering and Sturman (2009), we also tested a model wherein the items loaded both on their respective hypothesized latent constructs and on a common method factor. The results indicated that the average variance explained by the common method factor was only 11.20%. This is less than half of the amount of method variance (25%) that Williams, Cote and Buckley (1989) refer to for self-reported studies. This considerably reduces our concerns regarding this potential threat.

Despite these limitations, our research also has several strengths. The first is that we tested a mediation model investigated only once in the HPWS literature. Our study extends previous research focusing on the HPWS-employee’s work-related well-being relationships by proposing a more comprehensive model, including work-to-family interface. The second is that our study is in line with theories/models largely acknowledged in the literature (i.e., COR theory, Hobfoll, 1989, 2002; JD-R model, Schaufeli & Bakker, 2004; and enrichment process, Greenhaus & Powell, 2006).

That said, our study could be developed in two significant ways. As explained earlier, personal and job resources seem important in the relationships investigated. Various theories, detailed throughout our paper, support this idea—the COR theory (Hobfoll, 1989, 2002) with the gain/loss of resources; the enrichment process (Greenhaus & Powell, 2006) with the indirect (affective path) or direct (instrumental path) transfer of resources from one domain to another; and the JD-R model (Schaufeli & Bakker, 2004) with the motivational process. Therefore, to understand the underlying mechanism more fully, future research should investigate the role of personal and job resources in these relationships.

It would also be interesting to consider possible relevant variables that might influence the strength of relationships between organizational factors, such as HPWS and work-to-family interface, and their outcomes. Indeed, Greenhaus and Powell (2006) suggested that some factors could moderate the enrichment process. They claim an important moderator intervening in both the instrumental and affective paths is the *salience of Role B* (i.e., “the importance that individuals ascribe to roles played out in various domains such as work and family (Super, 1990; Super, Savickas, & Super, 1996)” (Cinamon, 2010, p. 85)). Greenhaus and Powell postulated that, even if workers have access to resources at work, they may not use these resources in the family because this domain may not be very salient for them and, therefore, not central to their lives. In the same vein, the importance accorded to work and family roles comprises a main predictor in the variance of conflict (Frone, 2003). WFC is intensified when work or family roles are salient and central to the person’s self-concept (Greenhaus & Beutell, 1985). Role salience seems, thus, to be a meaningful construct to consider in relation to work-family interface. However, very few studies have investigated the moderating effect of role salience within the work-to-family interface field (Lapierre, Kwan, Greenhaus, DiRenzo, & Shao, 2017).

### Practical implications

This study highlights the important role of HPWS in the development of employees’ well-being at work through their abilities to manage their work and family roles efficiently. By applying HPWS, managers provide their workers with greater access to resources through various practices. These practices tend to increase workers’ resources (e.g., enhancing their skills, increasing their motivation and facilitating their empowerment), allowing them to better manage their work and family lives and, thus, increasing their well-being at work.

Today, promoting employees’ health and well-being is necessary for organizations to survive in the competitive global environment. Therefore, considering the results of our study, it is important to implement such interconnected human resource practices throughout organizations. According to Price (2007, p. 55), “the Institute of Work Psychology (2001) at the University of Sheffield states that high performance work systems usually involve three main sets of management practices designed to enhance employee involvement, commitment and competencies:” “(1) changing the design and conduct of jobs”; “(2) ensuring that employees are given the knowledge and competences to handle high performance”; and “(3) resourcing and development practices designed to attract and keep the right people with the right motivation.” These practices are especially reflected in flexible work arrangements, teamwork, development of interpersonal skills, information sharing, guaranteeing of job security and sophisticated selection techniques. The most critical element of HPWS is support from top-level management. Indeed, HPWS are most often implemented by top managers (Becker & Huselid, 2006). Consequently, they have to be actively committed and involved in the implementation of HPWS (Gilbert, De Winne, & Sels, 2011), and understand and support such practices (Kroon, Van De Voorde, & Timmers, 2013).
